# Nanosilver induces a non-culturable but metabolically active state in *Pseudomonas aeruginosa*

**DOI:** 10.3389/fmicb.2015.00395

**Published:** 2015-05-05

**Authors:** Alexa M. Königs, Hans-Curt Flemming, Jost Wingender

**Affiliations:** Department of Aquatic Microbiology – Biofilm Centre, University Duisburg-Essen, EssenGermany

**Keywords:** silver nanoparticles, *Pseudomonas aeruginosa*, biofilms, non-culturable but metabolically active state, viability

## Abstract

The antimicrobial properties of silver nanoparticles (AgNPs) have raised expectations for the protection of medical devices and consumer products against biofilms. The effect of silver on bacteria is commonly determined by culture-dependent methods. It is as yet unknown if silver-exposed bacteria can enter a metabolically active but non-culturable state. In this study, the efficacy of chemically synthesized AgNPs and silver as silver nitrate (AgNO_3_) against planktonic cells and biofilms of *Pseudomonas aeruginosa* AdS was investigated in microtiter plate assays, using cultural as well as culture-independent methods. In liquid medium, AgNPs and AgNO_3_ inhibited both planktonic growth and biofilm formation. The efficacy of AgNPs and AgNO_3_ against established, 24 h-old biofilms and planktonic stationary-phase cells was compared by exposure to silver in deionized water. Loss of culturability of planktonic cells was always higher than that of the attached biofilms. However, resuspended biofilm cells became more susceptible to AgNPs and AgNO_3_ than attached biofilms. Thus, the physical state of bacteria within biofilms rendered them more tolerant to silver compared with the planktonic state. Silver-exposed cells that had become unculturable still displayed signs of viability: they contained rRNA, determined by fluorescent *in situ* hybridization, as an indicator for potential protein synthesis, maintained their membrane integrity as monitored by differential live/dead staining, and displayed significant levels of adenosine triphosphate. It was concluded that AgNPs and AgNO_3_ in concentrations at which culturability was inhibited, both planktonic and biofilm cells of *P. aeruginosa* were still intact and metabolically active, reminiscent of the viable but non-culturable state known to be induced in pathogenic bacteria in response to stress conditions. This observation is important for a realistic assessment of the antimicrobial properties of AgNPs.

## Introduction

The rise of antibiotic resistance in pathogenic bacteria is considered to be an emerging threat to human health and therefore of great concern (Fischbach and Walsh, 2009). Against this backdrop, there is ongoing intense search for alternatives, and the antimicrobial effect of silver has received increasing attention (Chernousova and Epple, 2013; Lemire et al., 2013). A recent application of silver as an antimicrobial agent is its use in the form of silver nanoparticles (AgNPs; Rai et al., 2009). Silver both as ions and as nanoparticles has found application in many consumer products, for example, in textiles or sprays and it is also increasingly used in cosmetics. In medical contexts, it has been proposed as a coating material for medical implants, catheters, wound dressings, instruments, and other applications for the prevention of infection (Chernousova and Epple, 2013). Multiple mechanisms have been assumed to underlie the antibacterial activity of AgNPs. Generally, the antimicrobial action of AgNPs is explained by the release of silver ions and their subsequent interaction with sulfhydryl groups of intracellular and membrane proteins as well as with DNA (Russell and Hugo, 1994; Sotiriou and Pratsinis, 2010). In addition, the generation of reactive oxygen species (ROS) has been reported in some studies to be involved in the antibacterial action of AgNPs. (Schierholz et al., 1998; Percival et al., 2005; Park et al., 2009; Cavalieri et al., 2014). Both effects may lead to alteration of protein structure and function with consequences such as disturbance of membrane permeability and respiration, and may result in inhibition of replication and transcription (Cabiscol et al., 2000; Holt and Bard, 2005). But there is also evidence that the direct interaction of nanoparticles with the bacterial cell contributes to the antimicrobial activity of AgNPs, with the nanoparticulate form and size itself playing an important role (Morones et al., 2005; Fabrega et al., 2009). Nanoparticles may attach to the cell surface, penetrate the cell membranes and thus initiate damage to the cell.

Bacterial biofilms are of particular concern in the clinical setting, because they can develop on medical devices and are involved in chronic human infections with painful and life-threatening consequences for the patients (Donlan and Costerton, 2002; Hall-Stoodley et al., 2004). Biofilms are accumulations of microorganisms that adhere to each other and/or to surfaces, and are typically surrounded by a matrix of self-produced extracellular polymeric substances (EPS; Hall-Stoodley et al., 2004). EPS mainly consist of polysaccharides, proteins, DNA and lipids; they determine the structural and functional integrity of microbial biofilms (Flemming and Wingender, 2010). In general, biofilm organisms display an increased tolerance to antimicrobials, including disinfectants, antibiotics, and toxic metal ions (Davies, 2003; Harrison et al., 2007). Anti-biofilm approaches employing AgNPs have been discussed (Markowska et al., 2013). AgNPs have been shown to be effective against biofilms of Gram-negative and Gram-positive pathogenic bacteria (Choi et al., 2010; Mohanty et al., 2012; Radzig et al., 2013; Gurunathan et al., 2014). Among the studies on AgNPs and biofilms, inactivation of established biofilms of *Escherichia coli* have been found to be four times (Choi et al., 2010) or 25 times (Radzig et al., 2013) more resistant to AgNPs than planktonic cells The production of EPS seems to be one of the factors conferring enhanced tolerance on biofilms against AgNPs (Kalishwaralal et al., 2010; Joshi et al., 2012).

Considering the antimicrobial action of silver, it has to be taken into account that the assessment of antimicrobial efficacy in the vast majority of publications is based on culture-dependent viable count methods. Microorganisms which do not grow on or in culture media are usually considered as dead or at least as finally inactivated. However, it has long been known that non-growing microorganisms do not necessarily have to be dead, but can still be metabolically active. Specifically, the phenomenon of bacteria that are alive, but fail to grow on standard laboratory media, on which they would normally grow, has been referred to as the viable but non-culturable (VBNC) state (Oliver, 2005, 2010; Li et al., 2014). The entry into this state is regarded as a survival strategy of bacteria in response to adverse environmental conditions such as unfavorable temperature, pH or nutrient conditions, or exposure to antimicrobial substances, irradiation, or desiccation (Oliver, 2010). The VBNC state is regarded as temporary and reversible, because resuscitation to the culturable state is possible under appropriate conditions (Oliver, 2010; Ramamurthy et al., 2014). At least some pathogenic bacteria retain their infectious potential in the VBNC state or can regain infectivity after resuscitation (Dwidjosiswojo et al., 2011; Li et al., 2014). In a recent review, Li et al. (2014) listed 85 bacterial species capable of entering the VBNC state, including 51 human pathogenic species including *Pseudomonas aeruginosa*. Of these pathogens, resuscitation has been reported in only 26 species.

Indicators of bacterial viability are respiratory activity, integrity of cytoplasmic membrane, maintenance of membrane potential, and the presence of mRNA or rRNA. Viability of non-culturable cells can be verified by a range of culture-independent methods, which allow the detection of cell integrity, physiological activity or the presence of nucleic acids, and include the determination of enzyme activities, respiratory activity, levels of adenosine triphosphate (ATP), membrane integrity as well as techniques based on fluorescence *in situ* hybridization (FISH), and polymerase chain reaction (PCR; Keer and Birch, 2003; Hammes et al., 2011; Rochelle et al., 2011; Li et al., 2014).

Among metals with an antimicrobial activity, copper ions have been shown to induce the VBNC state of the potential human pathogen *P. aeruginosa* (Dwidjosiswojo et al., 2011). When exposed to copper sulfate, the culturability of *P. aeruginosa* was completely inhibited, while total cell counts, the concentration of cells with an intact cytoplasmic membrane, and the number of cells with intact 16S rRNA, remained unchanged; *P. aeruginosa* was not cytotoxic in that state. When the copper stress was abolished by addition of the chelator diethyldithiocarbamate, complete restoration of culturability and cytotoxicity was observed within 14 days. The effect of AgNPs as well as silver ions from silver salts against planktonic cells and biofilms has usually been determined essentially by methods that are based on the detection of bacterial growth such as determination of plate counts, optical density measurements of planktonic cultures, or crystal violet staining of biofilm biomass (Kora and Arunachalam, 2011; Radzig et al., 2013; Gurunathan et al., 2014). Thus, it remained unknown if exposure to silver could also lead to a state of viable bacteria, in which they are not able to grow and are non-culturable, in analogy to the effect of copper.

In this study, the induction of a metabolically active but non-culturable state by exposure to silver from AgNPs and silver nitrate (AgNO_3_) was studied on planktonic cells and biofilms of *P. aeruginosa*. This organism is an opportunistic pathogen and a common cause of local and systemic as well as acute and chronic infections (Goodman et al., 2004; Mena and Gerba, 2009; Morita et al., 2014). This organism inherits an intrinsic resistance to a broad variety of antimicrobial substances (Poole, 2011). *P. aeruginosa* is one of the best studied biofilm organisms and is often involved in biofilm-associated infections which may become persistent and hard to treat such as chronic wound infections or lung infections of cystic fibrosis patients (Bjarnsholt et al., 2013). Microtiter plate assays were used to study the inhibitory activity of AgNPs in comparison to the silver salt AgNO_3_ (i) on the formation of *P. aeruginosa* biofilms, and (ii) on established biofilms. Both a culture method and culture-independent assays based on molecular techniques were employed in parallel to assess bacterial viability. Throughout this investigation, strain *P. aeruginosa* AdS was employed, because the phenomenon of the induction of a non-culturable but metabolically active state upon exposure to metal ions, specifically copper ions, had originally been discovered in this strain (Dwidjosiswojo et al., 2011).

## Materials and Methods

### Reagents

An aqueous dispersion of AgNPs (AgPURE^TM^ W 10) with a silver content of 10% (w/w) was provided by ras materials GmbH (Germany). The dispersion contained stabilizing agents, consisting of 4% (w/v) each of polyoxyethylene glycerol trioleate and polyoxyethylene (20) sorbitan monolaurate (Tween 20). The hydrodynamic diameter, the polydispersity index, and the zeta potential of AgNPs diluted in deionized water to final concentrations of 20 μg/ml Ag were determined by dynamic light scattering with a Malvern Zetasizer Nano ZS instrument (Malvern Instruments, UK). Transmission electron microscopy (TEM) was conducted with a CM 200 FEG instrument (Philips, The Netherlands). Particle diameter was measured manually, using the software Image J (Image Processing and Analysis in Java). Ultraviolet-visible (UV-VIS) absorbance spectra of silver nanoparticle dispersions diluted to a final concentration of 20 μg/ml Ag in deionized water or growth media were determined in the range between 350 and 750 nm, using a microplate reader (Tecan Infinite^®^ 200 PRO, Tecan Group, Switzerland). AgNO_3_ was purchased from Sigma–Aldrich (Germany).

### Bacteria and Growth Conditions

For all experiments, *P. aeruginosa* AdS was used; this strain was originally isolated from water of a plumbing system (Moritz et al., 2010). The strain was cultivated on LB agar (Lennox; Carl Roth GmbH, Germany) at 36°C for 24 h. A few colonies were inoculated in 5 ml LB broth (Lennox, Carl Roth GmbH, Germany) and the culture was incubated in a shaking water bath (GFL 1092, Gesellschaft für Labortechnik) at 180 rpm at 36°C for 18 h.

### Growth Curves

Silver nitrate solutions, AgNPs dispersions, and deionized water as a negative control (50 μl) were added to the wells of a 96-well microtiter plate with flat bottom (Brand, pureGrade^TM^ S) in triplicate. Concentrations of 0–10 μg/ml Ag for AgNO_3_ and 0–50 μg/ml Ag for AgNPs were employed. An overnight (18 h) culture of *P. aeruginosa* prepared as described above was diluted 50-fold in double concentrated LB broth and 50 μl were added to the wells. The plate was statically incubated at 36°C for 24 h in a microplate reader (Tecan Infinite^®^ 200 PRO) and the optical density at 570 nm was recorded every hour for 24 h to monitor the growth of the bacteria. Uninoculated LB broth was used as a sterility control. Due to the coloration of AgNPs, each AgNPs concentration was measured in LB medium without bacteria as a blank.

### Influence of Silver on Biofilm Formation

Silver nitrate solutions, AgNPs dispersions, and deionized water as a negative control (50 μl) were added to the wells of a 96-well microtiter plate with flat bottom (Brand, pureGrade^TM^ S) in octuplicate. Concentrations of 0–10 μg/ml Ag for AgNO_3_ solutions and 50 μg/ml Ag for AgNPs were employed. An overnight culture (18 h) of *P. aeruginosa* prepared as described above was diluted 50-fold in fresh LB broth and 50 μl of the suspension were added to the wells of the microtiter plate. The plate was statically incubated at 36°C for 24 h. As a negative control, deionized water was used and as a sterile control LB broth was used.

To stain the biofilms, planktonic bacteria were removed after 24 h by pipetting and discarding 90 μL with a multichannel pipette (Eppendorf Research). The biofilms attached to the walls of the well were washed twice with 200 μl of deionized water. The biofilm was stained by adding 125 μl of crystal violet solution (0.1%) to the wells. After incubation for 30 min at room temperature the crystal violet solution was removed and the wells were washed twice with 200 μl of deionized water. Subsequently, the plates were air-dried for at least 24 h. To solubilize the crystal violet from the biofilms, 200 μl of acetic acid (30%, v/v) were added to each well. The absorbance of the crystal violet solution was measured at 570 nm with a Tecan Infinite^®^ 200 PRO. This method was adapted from Stepanović et al. (2000).

In parallel the total cell counts and colony counts of silver-exposed planktonic and biofilm cells were determined. Ninety microliter of the planktonic bacteria were removed from the wells and an aliquot of 65 μl was transferred to a microcentrifuge tube (Sarstedt, 1.5 ml) containing 585 μl of deionized water in order to achieve a 10-fold dilution. The attached biofilms were washed twice with deionized water. Resuspension was performed by adding 100 μl of deionized water and scraping the biofilm off the well surfaces with a pipette tip. Sixty-five microliter of the suspension was transferred to a microcentrifuge tube (Sarstedt, 1.5 ml) containing 585 μl of deionized water. Silver was neutralized by adding 50 μl sodium thioglycolate (0.1%) and sodium thiosulfate (0.14%).

### Influence of Silver on Established Biofilms

An overnight (18 h) culture of *P. aeruginosa* prepared as described above was diluted 1:100 in fresh LB broth and 100 μl of the dilution was added to each well of a 96-well polystyrene microtiter plate with a flat bottom (pureGrade^TM^ S, Brand, Germany). Uninoculated LB broth was used as a sterile control.

After 24 h, the planktonic bacteria (90 μl) were removed by pipetting and were transferred to a microcentrifuge tube (Sarstedt, 1.5 ml). An aliquot of 65 μl was removed and transferred to a new microcentrifuge tube. Planktonic bacteria were harvested by centrifugation (10 min, 10477 × *g*, 18°C), washed twice in 200 μL deionized water, and finally resuspended in the equivalent test substance. In parallel the biofilms attached to the walls of the well were washed twice with 200 μl of deionized water. From a second microtiter plate the washed biofilms were suspended by scraping the biofilm off the well surface using the equivalent test substance. Planktonic bacteria, attached biofilms, and suspended biofilms were exposed to AgNO_3_ (100 μg/ml Ag), AgNPs (500 μg/ml), and deionized water as a control. The microtiter plates were statically incubated at 36°C for 24 h. Silver was neutralized using sodium thioglycolate (0.1%) and sodium thiosulfate (0.14%).

### Microscopic Determination of Total and Viable Cell Counts

The total cell count determination was carried out by staining the cells with 4′, 6-diamidino-2-phenylindole (DAPI). One milliliter DAPI solution [25 μg/ml in 2% (v/v) formaldehyde] was added to 4 ml of bacterial suspensions. After incubation in the dark at room temperature for 20 min, the solution was filtered through a black polycarbonate membrane filter (Millipore, 0.2 nm pore size). For determination of viable cells the LIVE/DEAD^®^ *Bac*Light bacterial viability kit (Molecular Probes) was used. 1.5 μl SYTO 9 and 1.5 μl propidium iodide, both dissolved in dimethyl sulfoxide (DMSO), were mixed. Propidium iodide was pre-diluted 1:200 in DMSO. Three microliter of the mixed stains was added to 1 ml of bacterial suspension. After incubation of the mixture in the dark at room temperature for 20 min, 4 ml sterile deionized water were added and the suspension was filtered through a black polycarbonate filter (Millipore, pore size 0.2 μm). Cells on membrane filters were enumerated under an epifluorescence microscope (Leitz, Laborlux S) at 1000-fold magnification with immersion oil (type N, Leica). Twenty randomly selected fields of view were examined for each filter with the help of a counting grid (100 μm × 100 μm).

### Determination of Colony Counts

Colony counts were determined by plating samples in duplicate on LB agar and incubation of the plates at 36°C for 24 h.

### FISH Analysis

Fluorescence *in situ* hybridization of *P. aeruginosa* cells was performed as described by Moritz et al. (2010), using probe Psae16S-182 labeled with Cy3 (Wellinghausen et al., 2005).

### Measurement of Adenosine Triphosphate (ATP) Concentrations

Adenosine triphosphate levels in bacterial suspensions were measured using the BacTiter-Glo^TM^ Microbial Cell Viability Assay (Promega) in combination with a GloMax^®^ 20/20 Luminometer (Promega) according to the manufacturer’s instructions.

### Statistical Analysis

Statistical significances were analyzed using analysis of variance (ANOVA) followed by a Dunnett’s test with a significance level of 5%. Statistical analysis was performed using the software R (http://www.r-project.org).

## Results

### Characterization of Nanoparticles

For all experiments, commercially available AgNPs were used. The hydrodynamic diameter of the AgNPs in deionized water was 35.6 nm, the polydispersity index was 0.5 and the zeta potential was – 3.7 mV. The average particle size measured by TEM was 6.7 ± 4.8 nm The UV-visible absorbance spectrum of the AgNPs (20 μg/ml) in deionized water revealed a distinct single peak with a maximum at 412 nm that corresponds to the surface plasmon resonance characteristic of AgNPs (for further information see Supplementary Material, Figures S1 and S2).

### Inhibitory Effect of Silver on Planktonic Growth and Biofilm Formation of *P. aeruginosa*

**FIGURE 1:**
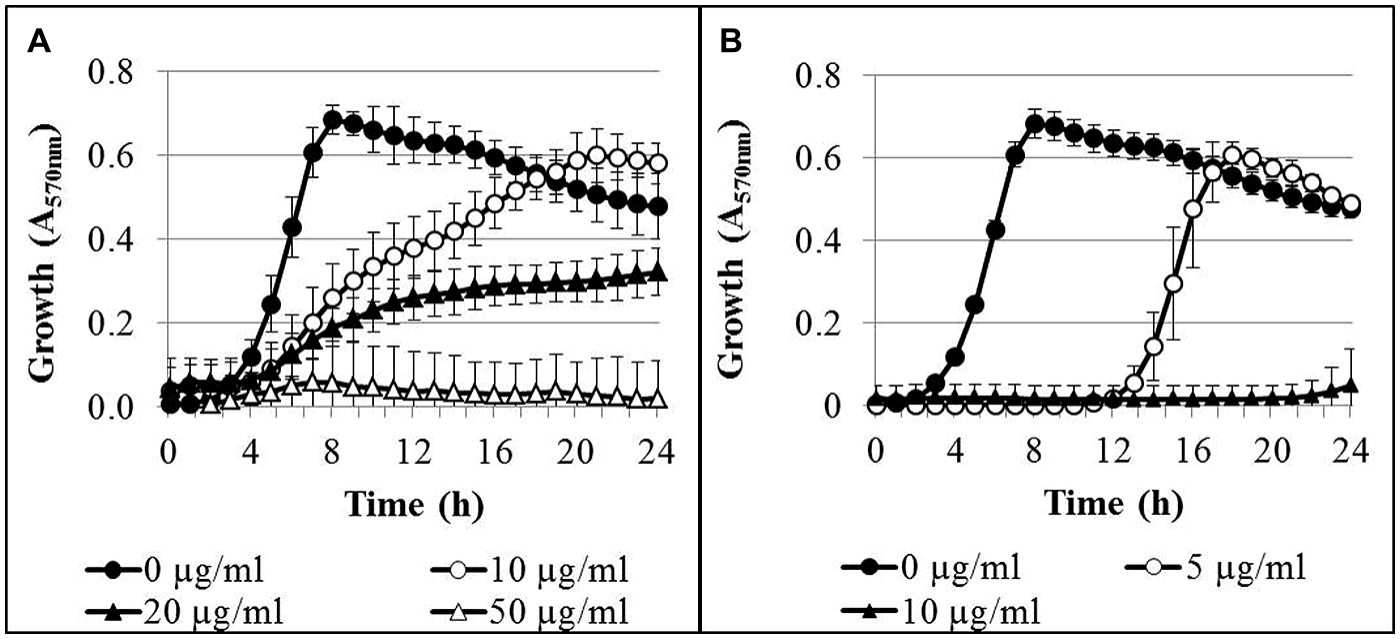
**Growth curves of *Pseudomonas aeruginosa* AdS in LB broth in the presence of different concentrations of AgNPs (A) and AgNO_3_ (B)**. The initial bacterial concentration was approximately 2 × 10^6^ cells/ml. *n* = 3.

The time-dependent growth inhibition in the presence of AgNPs and AgNO_3_ was determined in microtiter plates over 24 h. In control cultures without silver, the bacteria reached the stationary growth phase after 8 h of incubation (**Figure 1**). Addition of AgNPs to the bacterial cultures resulted in a concentration-dependent inhibition of bacterial growth (**Figure 1A**). At an AgNP concentration of 10 μg/ml Ag, delayed growth was observed, but after 18 h of incubation the optical density had reached that of the controls. Complete growth inhibition was reached at a concentration of 50 μg/ml Ag. In the presence of AgNO_3_, delayed growth of the bacteria was found at a concentration of 5 μg/ml Ag, and almost complete growth inhibition occurred at 10 μg/ml Ag (**Figure 1B**). It was verified that the AgNP matrix (composition of AgNP dispersion without silver) had no effect on the growth of *P. aeruginosa* at concentrations applied in the growth experiments (data not shown).

**FIGURE 2:**
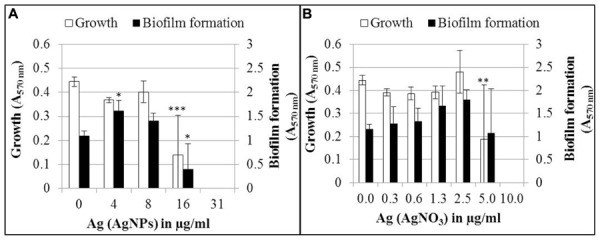
**Growth and biofilm formation of *P. aeruginosa* AdS in LB broth in the presence of different concentrations of AgNPs **(A)** and AgNO_3_ (B)**. Statistical analysis: ANOVA followed by Dunett’s test. Statistically significant (^∗^*p* < 0.05), very statistically significant (^∗∗^*p* < 0.01), highly statistically significant (^∗∗∗^*p* < 0.001), *n* = 3.

The effect of AgNPs and AgNO_3_ on biofilm formation was determined after 24 h growth in the wells of a microtiter plate (**Figure 2**). In this experiment, total cell growth was quantified by optical density (A_570__nm_), while biofilm biomass was quantified after removal of planktonic bacteria and staining the biofilm with crystal violet (A_570__nm_). Biofilm formation and planktonic growth were statistically significant (*p* < 0.05 and *p* < 0.001, respectively) inhibited by 16 μg/ml Ag for AgNPs (**Figure 2A**). Thirty-one microgram per milliliter Ag for AgNPs led to a complete inhibition of both planktonic growth and biofilm formation. Five microgram per milliliter Ag for AgNO_3_ inhibited the planktonic growth (*p* < 0.01), whereas inhibition of biofilm formation was statistically not significant (**Figure 2B**). A complete inhibition of both biofilm formation and planktonic growth was achieved at 10 μg/ml Ag. A tendency was observed that biofilm mass increased upon exposure to sub-inhibitory concentrations of AgNO_3_ and AgNPs, while no increase in cell density was observed. However, only the increase in biofilm mass at 4 μg/ml Ag for AgNPs was statistically significant (*p* < 0.05).

**FIGURE 3:**
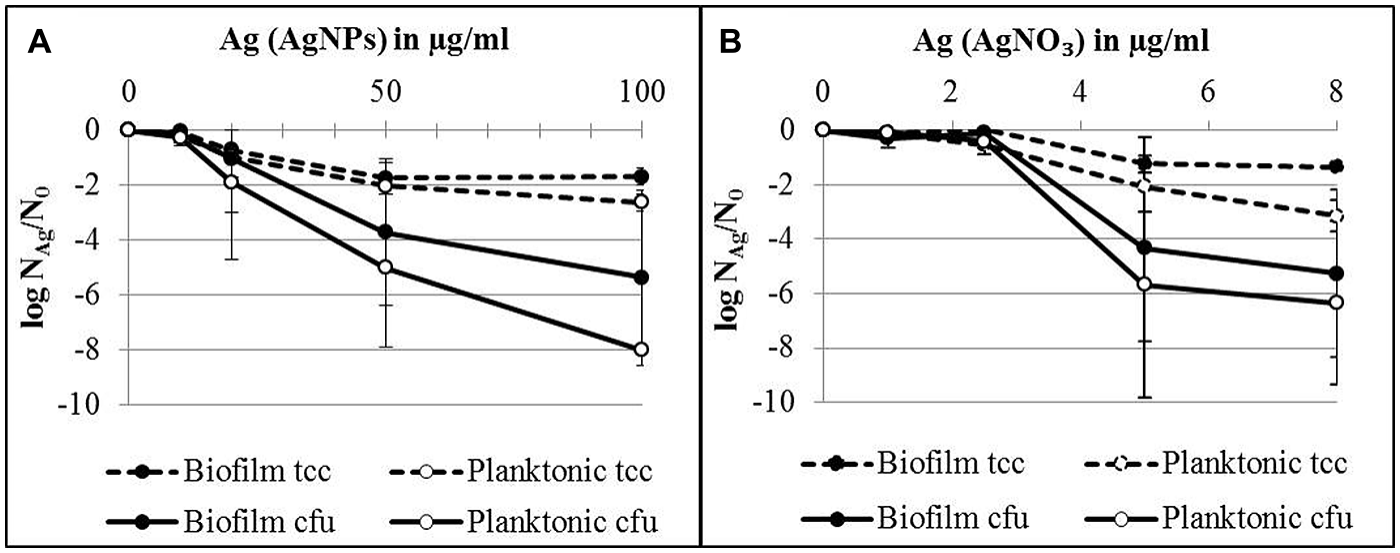
**Total cell counts (tcc) and colony counts (cfu) of planktonic cells and biofilms of *P. aeruginosa* AdS in the presence of AgNPs **(A)** and AgNO_3_**(B)** in LB broth after 24 h at 36°C.** N_0_ (planktonic cells): 2 × 10^9^ cells per well (tcc) and 1 × 10^8^ cfu per well, N_0_ (biofilm cells): 7 × 10^7^ cells per well (tcc) and 2 × 10^7^ cells/well. Statistical analysis: ANOVA followed by Dunett’s test. *n* = 3.

In order to quantify the effect of AgNPs and AgNO_3_ on planktonic bacteria and biofilm cells in more detail, planktonic bacteria and biofilm cells from the bacterial cultures were separated after silver exposure and the culturability and the total cell counts were determined. The results are expressed as logarithmic quotient of N_Ag_ (culturability or total cell counts of silver exposed cells) over N_0_ (culturability or total cell counts of the control without silver; **Figure 3**). Total cell number of the planktonic bacteria was inhibited with statistical significance at 20 μg/ml Ag for AgNPs (*p* < 0.01) and 2.5 μg/ml Ag for AgNO_3_ (*p* < 0.01). In contrast, the total cell number of biofilm cells was inhibited only at 50 μg/ml Ag for AgNPs (*p* < 0.01) and 5 μg/ml Ag for AgNO_3_ (*p* < 0.01). The percentage of culturable cells as a proportion of total cells of biofilm bacteria was significantly higher than that of planktonic cells (**Table 1**).

**Table 1:** Effect of silver nanoparticles (AgNPs) and silver nitrate (AgNO_**3**_) on the proportion of cfu to total cell counts of planktonic cells and biofilm cells of *P. aeruginosa* after growth for 24 h at 36^∘^C in LB medium.

Silver concentration (μg/ml)	Planktonic cells, percentage of cfu (%)	Biofilm cells, percentage of cfu (%)
**AgNPs**
0	5.10	30.03
10	4.61	21.22
20	6.62	59.35
50	0.54	7.82
100	<0.01	7.57
**AgNO_**3**_**
0	5.10	30.03
1	5.76	24.93
2.5	6.88	16.96
5	3.65	19.70
8	1.58	7.64

*Total cell counts correspond to 100%.*

### Effect of Silver on Established Biofilms

**FIGURE 4:**
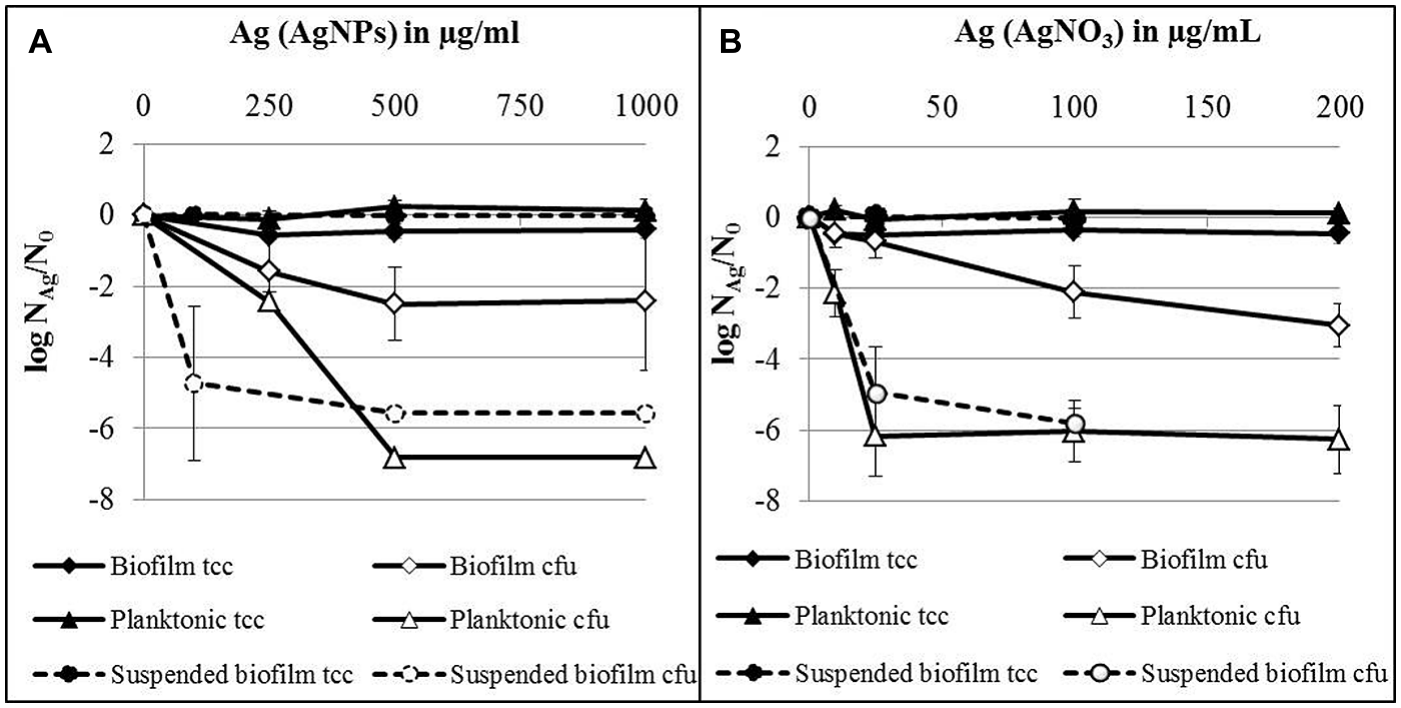
**Total cell counts (tcc) and colony counts (cfu) of established biofilms, suspended biofilms and planktonic bacteria of *P. aeruginosa* AdS in the presence of AgNPs **(A)** and AgNO_3_**(B)** in deionized water after 24 h at 36°C.** N_Ag_: tcc or cfu per well in the presence of silver, N_0_: tcc or cfu in the absence of silver. N_0_ (planktonic cells): 3.7 × 10^8^ cells per well (tcc) and 6.6 × 10^6^ cfu per well, N_0_ (biofilms): 1.5 × 10^8^ cells per well (tcc) and 1.2 × 10^6^ cfu per well, N_0_ (suspended biofilms): 3.9 × 10^7^ cells per well (tcc) and 5.3 × 10^6^ cfu per well. Statistical analysis: ANOVA followed by Dunett’s test. *n* = 2–7.

The influence of AgNPs and AgNO_3_ on established biofilms was compared with planktonic cells in the stationary growth phase, employing 24 h-old attached biofilms, suspended biofilms, and planktonic bacteria which were separately exposed to AgNPs or AgNO_3_ in deionized water. The results are expressed as logarithmic quotient of N_Ag_ (culturability or total cell counts of silver exposed cells) over N_0_ (culturability or total cell counts of the control without silver; **Figure 4**). For all tested silver concentrations (AgNPs and AgNO_3_) no statistically significant decrease of total cell counts was observed for planktonic bacteria, attached as well as suspended biofilms. Two hundred and fifty microgram per milliliter Ag for AgNPs caused a statistically significant effect on the concentration of culturable bacteria of both, planktonic bacteria (*p* < 0.05) and biofilms (*p* < 0.01; **Figure 4A**). Thousand microgram per milliliter Ag for AgNPs caused a maximal decline of the concentration of culturable biofilm bacteria by approximately 2.5 log units, while planktonic bacteria lost culturability completely at 500 μg/ml Ag (decrease by seven log units). In parallel, biofilm bacteria were resuspended during the addition of silver. AgNPs with 100 μg/ml Ag statistically significant (*p* < 0.05) reduced the culturability of *P. aeruginosa* by about 5 log units. In the case of AgNO_3_, a statistically significant effect on the culturability was observed at 25 μg/ml Ag (**Figure 4B**) for both planktonic bacteria (*p* < 0.001) and biofilm cells (*p* < 0.01). At this concentration, planktonic cells already revealed maximal loss of culturability by 6 log units, whereas a maximal decrease of culturability of biofilm bacteria by 3 log units (*p* < 0.01) was observed at 200 μg/ml Ag (**Figure 4B**). Twenty-five microgram per milliliter Ag from AgNO_3_ led to almost complete inhibition of (*p* < 0.05) the culturability of suspended biofilm cells (decrease by 5 log units).

In summary, silver from AgNO_3_ was considerably more effective against *P. aeruginosa* than silver from nanoparticles. Planktonic bacteria were more susceptible toward silver, independent of its source (AgNPs or AgNO_3_), than attached biofilms. Biofilm cells became more susceptible toward silver, after they were suspended.

### Viability of Silver-Exposed *P. aeruginosa*

**FIGURE 5:**
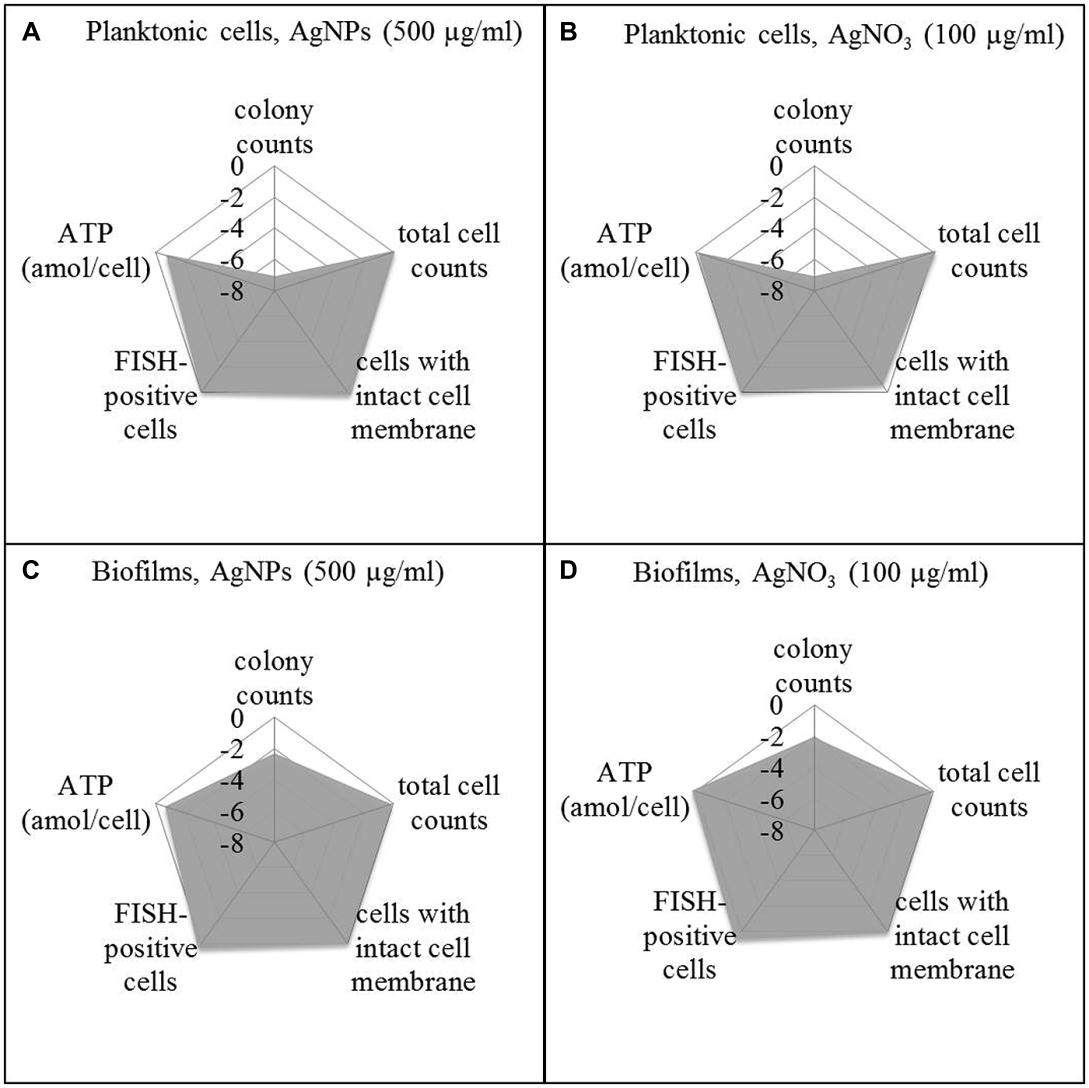
**Viability pattern of planktonic cells **(A,B)** and biofilms **(C,D)** of *P. aeruginosa* AdS, exposure to 500 μg/ml AgNPs **(A,C)**, exposure to 100 μg/ml AgNO_3_ (B,D).** Axis: log N_Ag_/N_0_. N_Ag_: viability parameter in the presence of silver, N_0_: viability parameter in the absence of silver. N_0_: 1.67 × 10^7^ cfu per well, 7.6 × 10^8^ cells per well (total cell count), 1.4 × 10^7^ membrane intact cells (LIVE/DEAD), 4.0 × 10^8^ cells per well (FISH), 0.018 ATP (amol/cell), for details see: Supplementary Material Table S1. Statistical analysis: ANOVA followed by Dunett’s test. *n* = 3.

In order to offer a better overview on the viability data, they were standardized on the basis of the corresponding control as log N_Ag_ (viability parameter of silver exposed cells) divided by log N_0_ (viability parameter of control without silver; **Figure 5**) and presented as spiderweb diagrams. This was considered a way to combine data from various experiments for comparison_._ Original data from Figures 6 and 7 are provided in the Supplementary Material (Tables S1 and S2). Five-hundred microgram per milliliter Ag for AgNPs and 100 μg/ml led to a statistically significant decrease of the concentration of culturable planktonic cells by six oders of magnitude (*p* < 0.001, **Figures 5A,B**) and of biofilm cells by one order of magnitude (*p* < 0.01, **Figures 5C,D**), whereas the total cell counts did not show a statistically significant change.

To distinguish between viable and dead cells, the LIVE/DEAD *Bac*Light Bacterial Viability Kit was employed. Using a mixture of SYTO 9 and propidium iodide, bacteria with intact membranes display green fluorescence, whereas bacteria with damaged membranes cannot reject propidium iodide which then binds to nucleic acids and causes red fluorescence. Membrane damage is interpreted as a sign of cell death. Thus, using a combination of SYTO 9 and propidium iodide allows distinction between live and injured or dead bacterial cells. It was found that exposure of *P. aeruginosa* (both planktonic bacteria and biofilms) to AgNPs and AgNO_3_ revealed no statistically significant change in the level of green fluorescent cells, indicating that AgNPs and AgNO_3_ did not impair membrane integrity of the bacteria (**Figure 5**). In parallel, the FISH method was used to quantify the concentration of bacteria with intact rRNA as an indicator of metabolic activity. Using *P. aeruginosa*-specific oligonucleotide probe Psae16S-182 targeted at 16S rRNA, no statistically significant decrease of the concentration of FISH-positive cells occurred upon exposure to AgNPs and AgNO_3_ (**Figure 5**). As an additional potential indicator of cell viability, ATP was determined in the bacterial suspensions exposed to AgNPs and AgNO_3_ and was present at all silver concentrations. Five hundred microgram per milliliter Ag for AgNPs led to a statistically significant (*p* < 0.05) decrease of the ATP concentration in planktonic bacteria (about 75%), while the ATP level in the biofilm cells remained stable upon AgNP exposure. AgNO_3_ exposure did not show any statistically significant decrease of the ATP levels of *P. aeruginosa* (**Figure 5**).

After silver exposure, microscopy demonstrated that the cells remained intact (not shown), which indicated that no lysis had occurred. Membrane integrity was maintained, as demonstrated by exclusion of propidium iodide. rRNA also was still present to the same extent as in the control. The ATP levels remained stable, except for planktonic bacteria exposed to AgNPs (500 μg/ml) Ag.

## Discussion

The goal of this study was to determine if AgNPs or AgNO_3_ actually kill bacteria or only inhibit their growth, and if the bacteria are protected when they live in biofilms. *P. aeruginosa* AdS, grown in complex growth medium (LB), was employed to investigate the effect of chemically synthesized AgNPs in comparison to Ag of AgNO_3_. The experiments were carried out in 96-well microtiter plates. A concentration-dependent growth inhibition was observed in liquid media. This confirms previous studies reporting growth inhibition of *P. aeruginosa* in complex liquid media or on agar media by other types of AgNPs. To some extent, comparison is hampered by the fact that the AgNPs differed in their properties such as mode of synthesis (chemically produced and biogenic), size and shape as well as in the presence of stabilizing agents (Kora and Arunachalam, 2011; Sintubin et al., 2011; Mohanty et al., 2012; Radzig et al., 2013). This may partially explain why literature data of minimal inhibitory concentrations (MIC) of AgNPs and silver ions for different *P. aeruginosa* strains vary over quite a large range. For example, Martinez-Gutierrez et al. (2010) determined a MIC of 0.4 μg/ml for AgNPs (chemically synthesized, 20–25 nm) whereas Sintubin et al. (2011) observed a MIC of 500 μg/ml for AgNPs (chemically synthesized 20–25 nm).

One of the mechanisms proposed to be involved in the antibacterial effect of AgNPs considers silver ions, released from the nanoparticles, as the effective agents (Morones et al., 2005; Sintubin et al., 2011). Priester et al. (2014) showed that toxicity of silver was associated to ROS and cell membrane damage; in their study, toxicity of AgNPs was due to Ag^+^ ions, not AgNps. In our study, calculated on the basis of the concentration of elemental silver, Ag from AgNO_3_ exhibited considerably higher growth inhibition of *P. aeruginosa* than silver from AgNPs (**Figure 1**). Retarded release of silver ions in combination with their interaction with components in the medium may have resulted in lower concentrations of free silver ions, available for antibacterial activity than in the presence of AgNO_3_ (Grade et al., 2012; Loza et al., 2014). In contrast, some other studies found lower values of minimum inhibitory concentrations for AgNO_3_ compared to AgNPs, pointing to a higher antibacterial effect of AgNO_3_ on growth of *P. aeruginosa* (Sintubin et al., 2011; Radzig et al., 2013). Mohanty et al. (2012) reported that starch-stabilized AgNPs were more effective in the growth inhibition of *P. aeruginosa* than AgNO_3_. This could not be confirmed for the AgNPs used in our study.

In order to identify a possible protective effect of the biofilm mode of growth, the tests included both planktonic and biofilm cells. AgNPs used in the current study as well as AgNO_3_ were also shown to inhibit biofilm formation of strain *P. aeruginosa* AdS. This finding confirms results of studies in which inhibition of biofilm formation by AgNPs and AgNO_3_ was also found for other strains of *P. aeruginosa*. Kalishwaralal et al. (2010) reported inhibition of *P. aeruginosa* biofilm formation by biologically synthesized AgNPs in 96-well polystyrene tissue culture plates during incubation of the bacteria in brain heart infusion broth at 37°C for 24 h, with complete inhibition in the presence of 100 nM (10.8 ng/ml) AgNPs. Mohanty et al. (2012) described inhibition of *P. aeruginosa* biofilm formation by starch-stabilized AgNPs. At concentrations of 1 and 2 μM AgNPs, biofilm formation was decreased by 50 and 85%, respectively, during incubation of the bacteria in Luria-Bertani broth in polystyrene microtiter plates at 37°C for 24 h. Radzig et al. (2009) showed inhibition of the biofilm formation of *P. aeruginosa* by AgNO_3_ (0.6 μgmL^-1^ Ag).

With regard to already established biofilms, there are only very few studies in which the effect of AgNPs and silver salts was investigated. In our study, silver was able to inhibit growth of established biofilms and planktonic bacteria in the stationary growth phase. Two-four hours-old biofilms and planktonic bacteria were separately exposed to AgNPs or AgNO_3_ in deionized water for 24 h. Silver (AgNPs and AgNO_3_) led to a decrease of culturability of established biofilms and planktonic bacteria. The concentrations to achieve this were higher than needed for the inhibition of biofilm formation, although the exposure was carried out in deionized water without possible quenching effects of medium components. Planktonic bacteria were more susceptible toward both AgNPs and AgNO_3_ than biofilm cells. This clearly indicates the protective effect of the biofilm mode of life. Choi et al. (2010) showed that for the inactivation of established biofilms of *E. coli*, a fourfold higher concentration was required than for planktonic bacteria. In contrast, Radzig et al. (2013) observed an almost complete reduction of the number of membrane-intact cells of *E. coli* AB1157 biofilms grown on glass as determined by live/dead staining. However, it is well known that biofilms are considerably more tolerant to antimicrobial agents than planktonic cells (Stewart and Costerton, 2001; Davies, 2003). This also applies to toxic metals (Booth et al., 2011). Biofilms can bind ions (Leis and Flemming, 2002) either to EPS or cell walls, and can thus attenuate the toxic effects. Joshi et al. (2012) showed that EPS protected *E. coli* from AgNPs and plausibly supposed that the protective effect may be attributed to aggregation of the nanoparticles in the biofilm matrix; Choi et al. (2010) used the same explanation when they found lower efficacy of AgNPs toward biofilms. Another protective effect may occur in anaerobic areas of biofilms. Xiu et al. (2012) report that AgNPs do not release silver ions under anaerobic conditions. It is well known that such conditions can commonly occur within biofilms. The existence of anaerobic microdomains in biofilms is well known (Lawrence et al., 2007). The protective effect of the biofilm as a whole could be clearly demonstrated with resuspended biofilm cells in this study. After resuspension, these cells were equally susceptible to AgNPs and AgNO_3_ as planktonic cells (see dotted lines in **Figures 4** and **5**). Obviously, the protective effect of biofilms is connected to the situation of the biofilm cells, embedded in the EPS matrix. A similar effect of the biofilm has been reported by Said et al. (2014) when the biofilm was disrupted by ethylenediaminetetraacetic acid (EDTA) and benzethonium chloride (BC): after disintegration of the biofilm matrix, the biofilm cells became much more susceptible to AgNPs, as determined by microcalorimetry. Control experiments showed that neither EDTA nor BC alone had a bactericidal effect. The authors conclude that it was the synergistic action of EDTA and BC disrupting the biofilm with silver being bactericidal that leads to efficacy of AgNPs. However, other viability parameters such as membrane integrity were not determined.

Interestingly, at subinhibitory concentrations (4 μg/ml AgNPs), biofilm mass increased, as determined by staining with crystal violet, while the overall cell density did not. As crystal violet stains not only cells but also EPS, the increase in biofilm mass is likely due to an increase of EPS and might be interpreted as a stress response to sub-lethal silver concentrations. With the same type of assay, a similar trend of enhanced formation of biofilm mass was shown for *P. aeruginosa* and other Gram-negative and Gram-positive bacteria in the presence of AgNO_3_ (Radzig et al., 2009) and AgNPs (Shahrokh and Emtiazi, 2009). Thus, it has to be taken into account that sub-lethal concentrations of silver can induce or enhance biofilm formation probably by EPS production. This might lead to further insusceptibility of the bacteria toward biocides, because they are protected within the biofilm (Stewart and Costerton, 2001; Davies, 2003) which was clearly confirmed by our study.

What is also of interest is that exposure to silver led only to a decrease of the number of culturable cells but not of total cell numbers, indicating that no cell lysis has occurred (**Figures 4A,B**). This observation led to the question if the non-growing moiety might not have been actually killed or has rather entered a metabolically non-culturable state, a characteristic aspect of the VBNC phenomenon (Oliver, 2005; Li et al., 2014). In order to address this question, culture-independent methods were applied. A range of such methods is available (Hammes et al., 2011). In our study, we included (i) testing for the existence of intact cell membranes, which is commonly interpreted as a viability sign, detected by the LIVE/DEAD system, (ii) application of FISH, providing information about the presence of rRNA as an indicator of possible active protein production, and (iii) determining the ATP concentration as indicator of the energy status of cells. These data were compared to the total cell numbers and the numbers of colony forming cells. If the cells are really dead upon silver exposure, they should not only stop growing. Above that, they should not contain significant amounts of rRNA, the membranes should be damaged, and the energy status should be low to zero. In order to arrange the results more clearly in comparison to the culturability data, they were presented in **Figure 5** as spiderweb diagrams on the basis of N/N_0_. They clearly reveal the drastic decrease of the numbers of colony-forming units of planktonic cells, both upon AgNP and AgNO_3_ exposure. However, spiderweb pattern clearly reveals that no lysis has occurred (total cell counts remained constant), the membranes remained intact, the signal for rRNA is the same as that of the control; only the ATP content decreased by up to 75% with a significant amount of cellular energy still remaining. These parameters indicate that the cells have not been killed by silver ions or nanoparticles but rather have entered a metabolically active but non-culturable state which might be interpreted as VBNC. There is still a debate if the return to the culturable state is necessary to meet the definition of the VBNC state. In their excellent review, Li et al. (2014) did not include reversal to culturability into the definition. They reported a VBNC state of 51 human pathogens with only 26 of them resuscitated. The resuscitation conditions vary considerably. It has to be expected that the number of resuscitated species from this list will increase with further research on the VBNC state. In the case of *P. aeruginosa*, resuscitation remains a realistic option, although in this study, the conditions for resuscitation could not yet be elaborated. This option should be considered in the assessment of the antibacterial efficacy of AgNPs and as a possible reason if failures in the antimicrobial efficacy of AgNPs occur.

## Conflict of Interest Statement

The authors declare that the research was conducted in the absence of any commercial or financial relationships that could be construed as a potential conflict of interest.

## References

[B1] Bjarnsholt T. Alhede M. Alhede M. Eickhardt-Sørensen S. R. Moser C. Kühl M. (2013). The in vivo biofilm. *Trends Microbiol.* 21 466–474 10.1016/j.tim.2013.06.002 23827084

[B2] Booth S. C. Workentine M. L. Wen J. Shaykhutdinov R. Vogel H. J. Ceri H. (2011). Differences in metabolism between the biofilm and planktonic response to metal stress. *J. Proteome Res.* 10 3190–3199 10.1021/pr2002353 21561166

[B3] Cabiscol E. Tamarit J. Ros J. (2000). Oxidative stress in bacteria and protein damage by reactive oxygen species. *Int. Microbiol.* 3 3–8. 10963327

[B4] Cavalieri F. Tortora M. Stringaro A. Colone M. Baldassarri L. (2014). Nanomedicines for antimicrobial interventions. *J. Hosp. Infect.* 88 183–190 10.1016/j.jhin.2014.09.009 25447199

[B5] Chernousova S. Epple M. (2013). Silver as antibacterial agent: ion, nanoparticle, and metal. *Angew. Chem. Int. Ed.* 52 1636–1653 10.1002/anie.201205923 23255416

[B6] Choi O. Yu C.-P. Esteban Fernández G. Hu Z. (2010). Interactions of nanosilver with *Escherichia coli* cells in planktonic and biofilm cultures. *Water Res.* 44 6095–6103 10.1016/j.watres.2010.06.069 20659752

[B7] Davies D. (2003). Understanding biofilm resistance to antibacterial agents. *Nat. Rev. Microbiol.* 2 114–122 10.1038/nrd1008 12563302

[B8] Donlan R. M. Costerton J. W. (2002). Biofilms: survival mechanisms of clinically relevantmicroorganisms. *Clin. Microbiol. Rev*. 15 167–193 10.1128/CMR.15.2.167-193.2002 11932229 PMC118068

[B9] Dwidjosiswojo Z. Richard J. Moritz M. M. Dopp E. Flemming H.-C. Wingender J. (2011). Influence of copper ions on the viability and cytotoxicity of *Pseudomonas aeruginosa* under conditions relevant to drinking water environments. *Int. J. Hyg. Environ. Health* 214 485–492 10.1016/j.ijheh.2011.06.004 21742552

[B10] Fabrega J. Fawcett S. R. Renshaw C. Lead J. R. (2009). Silver nanoparticle impact on bacterial growth: effect of pH, concentration, and organic matter. *Environ. Sci. Technol.* 43 7285–7290 10.1021/es803259g 19848135

[B11] Fischbach M. A. Walsh C. T. (2009). Antibiotics for emerging pathogens. *Science* 325 1089–1093 10.1126/science.1176667 19713519 PMC2802854

[B12] Flemming H.-C. Wingender J. (2010). The biofilm matrix. *Nat. Rev. Microbiol.* 8 623–633 10.1038/nrmicro2415 20676145

[B13] Goodman A. L. Kulasekara B. Rietsch A. Boyd D. Smith R. S. Lory S. (2004). A signaling network reciprocally regulates genes associated with acute infection and chronic persistence in *Pseudomonas aeruginosa*. *Dev. Cell* 7 745–754 10.1016/j.devcel.2004.08.020 15525535

[B14] Grade S. Eberhard J. Neumeister A. Wagener P. Winkel A. Stiesch M. (2012). Serum albumin reduces the antibacterial and cytotoxic effects of hydrogel-embedded colloidal silver nanoparticles. *RSC Adv.* 2 7190–7196 10.1039/C2RA20546G

[B15] Gurunathan S. Han J. W. Kwon D.-H. Kim J.-H. (2014). Enhanced antibacterial and antibiofilm activities of silver nanoparticles against Gram-negative and Gram-positive bacteria. *Nanoscale Res. Lett.* 9:373 10.1186/1556-276X-9-373 PMC4127560 25136281

[B16] Hall-Stoodley L. Costerton J. W. Stoodley P. (2004). Bacterial biofilms: from the natural environment to infectious diseases. *Nat. Rev. Micobiol.* 2 95–108 10.1038/nrmicro821 15040259

[B17] Hammes F. Berney M. Egli T. (2011). Cultivation-independent assessment of bacterial viability. *Adv. Biochem. Eng. Biotechnol.* 124 123–150 10.1007/10_2010_95 21069588

[B18] Harrison J. J. Ceri H. Turner R. J. (2007). Multimetal resistance and tolerance in microbial biofilms. *Nat. Rev. Microbiol.* 5 928–938 10.1038/nrmicro1774 17940533

[B19] Holt K. B. Bard A. J. (2005). Interaction of silver(I) ions with the respiratory chain of *Escherichia coli*: an electrochemical and scanning electrochemical microscopy study of the antimicrobial mechanism of micromolar Ag^+^. *Biochemistry* 44 13214–13223 10.1021/bi0508542 16185089

[B20] Joshi N. Ngwenya B. T. French C. E. (2012). Enhanced resistance to nanoparticle toxicity is conferred by overproduction of extracellular polymeric substances. *J. Hazard. Mater.* 241–242 363–370 10.1016/j.jhazmat.2012.09.057 23098996

[B21] Kalishwaralal K. BarathManiKanth S. Pandian S. R. Deepak V. Gurunathan S. (2010). Silver nanoparticles impede the biofilm formation by *Pseudomonas aeruginosa* and *Staphylococcus epidermidis*. *Colloids Surf. B Biointerfaces* 79 340–344 10.1016/j.colsurfb.2010.04.014 20493674

[B22] Keer J. T. Birch L. (2003). Molecular methods for the assessment of bacterial viability. *J. Microbiol. Methods* 53 175–183 10.1016/S0167-7012(03)00025-3 12654489

[B23] Kora A. J. Arunachalam J. (2011). Assessment of antibacterial activity of silver nanoparticles on *Pseudomonas aeruginosa* and its mechanism of action. *World J. Microbiol. Biotechnol.* 27 1209–1216 10.1007/s11274-010-0569-2

[B24] Lawrence J. R. Swerhone G. D. Kuhlicke U. Neu T. (2007). In-situ evidence for microdomains in the polymer matrix of bacterial microcolonies. *Can. J. Microbiol*. 53 450–458 10.1139/W06-146 17538657

[B25] Leis A. Flemming H.-C. (2002). “Activity and carbon transformations in biofilms,” in *Biofilms: Encyclopedia of Environmental Microbiology* Vol. 1 eds Flemming H.-C. Bitton G. (New York, NY: Wiley-Interscience) 81–92.

[B26] Lemire J. A. Harrison J. J. Turner R. J. (2013). Antimicrobial activity of metals: mechanisms, molecular targets and applications. *Nat. Rev. Microbiol.* 11 371–384 10.1038/nrmicro3028 23669886

[B27] Li L. Mendis N. Trigui H. Oliver J. D. Faucher S. P. (2014). The importance of the viable but non-culturable state in human bacterial pathogens. *Front. Microbiol.* 5:258 10.3389/fmicb.2014.00258 PMC4040921 24917854

[B28] Loza K. Diendorf J. Sengstock C. Ruiz-Gonzalez L. Gonzalez-Calbet J. M. Vallet-Regi M. (2014). The dissolution and biological effects of silver nanoparticles in biological media. *J. Mater. Chem. B* 2 1634–1643 10.1039/c3tb21569e 32261391

[B29] Markowska K. Grudniak A. M. Wolska K. I. (2013). Silver nanoparticles as an alternative strategy against bacterial biofilms. *Acta Biochim. Pol.* 60 523–530. 24432308

[B30] Martinez-Gutierrez F. Olive P. L. Banuelos A. Orrantia E. Nino N. Sanchez E. M. (2010). Synthesis, characterization, and evaluation of antimicrobial and cytotoxic effect of silver and titanium nanoparticles. *Nanomedicine* 6 681–688 10.1016/j.nano.2010.02.001 20215045

[B31] Mena K. D. Gerba C. P. (2009). Risk assessment of *Pseudomonas aeruginosa* in water. *Rev. Environ. Contam. Toxicol.* 201 71–115 10.1007/978-1-4419-0032-6_3 19484589

[B32] Mohanty S. Mishra S. Jena P. Jacob B. Sarkar B. Sonawane A. (2012). An investigation on the antibacterial, cytotoxic, and antibiofilm efficacy of starch-stabilized silver nanoparticles. *Nanomedicine* 8 916–924 10.1016/j.nano.2011.11.007 22115597

[B33] Morita Y. Tomida J. Kawamura Y. (2014). Responses of *Pseudomonas aeruginosa* to antimicrobials. *Front. Microbiol.* 4:422 10.3389/fmicb.2013.00422 PMC3884212 24409175

[B34] Moritz M. M. Flemming H.-C. Wingender J. (2010). Integration of *Pseudomonas aeruginosa* and *Legionella pneumophila* in drinking water biofilms grown on domestic plumbing materials. *Int. J. Hyg. Environ. Health* 213 190–197 10.1016/j.ijheh.2010.05.003 20556878

[B35] Morones J. R. Elechiguerra J. L. Camacho A. Holt K. Kouri J. B. Ramírez J. T. (2005). The bactericidal effect of silver nanoparticles. *Nanotechnology* 16 2346–2353 10.1088/0957-4484/16/10/059 20818017

[B36] Oliver J. D. (2005). The viable but non-culturable state in bacteria. *J. Microbiol.* 43 93–100 10.3389/fmicb.2014.00258 15765062

[B37] Oliver J. D. (2010). Recent findings on the viable but non-culturable state in pathogenic bacteria. *FEMS Microbiol. Rev.* 34 415–425 10.1111/j.1574-6976.2009.00200 20059548

[B38] Park H.-J. Kim J. Y. Kim J. Lee J.-H. Hahn J.-S. Gu M. B. (2009). Silver-ion- mediated reactive oxygen species generation affecting bactericidal activity. *Water Res.* 43 1027–1032 10.1016/j.watres.2008.12.002 19073336

[B39] Percival S. L. Bowler P. G. Russel D. (2005). Bacterial resistance to silver in wound care. *J. Hosp. Infect.* 60 1–7 10.1016/j.jhin.2004.11.014 15823649

[B40] Poole K. (2011). Pseudomonas aeruginosa: resistance to the max. *Front. Microbiol.* 2:65 10.3389/fmicb.2011.00065 PMC3128976 21747788

[B41] Priester J. H. Singhal A. Wu B. Stucky G. D. Holden P. A. (2014). Integrated approach to evaluating the toxicity of novel cysteine-capped silver nanoparticles to *Escherichia coli* and *Pseudomonas aeruginosa*. *Analyst* 139 954–963 10.1039/c3an01648j 24343373

[B42] Radzig M. A. Koksharova O. A. Khmel I. A. (2009). Antibacterial effects of silver ions on growth of gram-negative bacteria and biofilm formation. *Mol. Genet. Microbiol. Virol.* 24 194–199 10.3103/S0891416809040065 20017360

[B43] Radzig M. A. Nadtochenko V. A. Koksharova O. A. Kiwi J. Lipasova V. A. Khmel I. A. (2013). Antibacterial effects of silver nanoparticles on gram-negative bacteria: influence on the growth and biofilms formation, mechanisms of action. *Colloids Surf. B Biointerfaces* 102 300–306 10.1016/j.colsurfb.2012.07.039 23006569

[B44] Rai M. Yadav A. Gade A. (2009). Silver nanoparticles as a new generation of antimicrobials. *Biotechnol. Adv.* 27 76–83 10.1016/j.biotechadv.2008.09.002 18854209

[B45] Ramamurthy T. Ghosh A. Pazhani G. P. Shinoda S. (2014). Current perspectives on viable but non-culturable (VBNC) pathogenic bacteria. *Front. Public Health* 2:103 10.3389/fpubh.2014.00103 PMC4116801 25133139

[B46] Rochelle P. A. Camper A. K. Nocker A. Burr M. (2011). “Are they alive? Detection of viable organisms and functional gene expression using molecular techniques,” in *Environmental Microbiology* eds Sen K. Ashbolt N. (Norfolk: Caister Acad. Press) 179–202.

[B47] Russell A. D. Hugo W. B. (1994). Interaction of silver nitrate with readily identifiable groups: relationship to the antibacterial action of silver ions. *Prog. Med. Chem*. 31 351–370 10.1016/S0079-6468(08)70024-9 8029478

[B48] Said J. Dodoo C. C. Walker M. Parsons D. Stapleton P. Beezer A. E. (2014). An in vitro test of the efficacy of silver-containing wound dressings against *Staphylococcus aureus* and *Pseudomonas aeruginosa* in simulated wound fluid. *Int. J. Pharm.* 28 123–128 10.1016/j.ijpharm.2013.12.037 24374221

[B49] Schierholz J. M. Lucas L. J. Rump A. Pulverer G. (1998). Efficacy of silver-coated medical devices. *J. Hosp. Infect.* 40 257–262 10.1016/S0195-6701(98)90301-2 9868616

[B50] Shahrokh S. Emtiazi G. (2009). Toxicity and unusual biological behavior of nanosilver on gram positive and negative bacteria assayed by microtiter-plate. *Eur. J. Biol. Sci.* 1 28–31.

[B51] Sintubin L. de Gusseme B. van der Meeren P. Pycke B. F. Verstraete W. Boon N. (2011). The antibacterial activity of biogenic silver and its mode of action. *Appl. Microbiol. Biotechnol.* 91 153–162 10.1007/s00253-011-3225-3 21468709

[B52] Sotiriou G. A. Pratsinis S. E. (2010). Antibacterial activity of nanosilver ions and particles. *Environ. Sci. Technol.* 44 5649–5654 10.1021/es101072s 20583805

[B53] Stepanović S. Vuković D. Dakić I. Savić B. Švabić-Vlahović M. (2000). A modified microtiter-plate test for quantification of staphylococcal biofilm formation. *J. Microbiol. Methods* 40 175–179 10.1016/S0167-7012(00)00122-6 10699673

[B54] Stewart P. S. Costerton J. W. (2001). Antibiotic resistance of bacteria in biofilms. *Lancet* 358 135–138 10.1016/S0140-6736(01)05321-1 11463434

[B55] Wellinghausen N. Köthe J. Wirths B. Sigge A. Poppert S. (2005). Superiority of molecular techniques for identification of gram-negative, oxidase-positive rods, including morphologically non-typical *Pseudomonas aeruginosa*, from patients with cystic fibrosis. *J. Clin. Microbiol.* 43 4070–4075 10.1128/JCM.43.8.4070-4075.2005 16081953 PMC1233906

[B56] Xiu Z. M. Zhang Q. B. Puppala H. L. Colvin V. L. Alvarez P. J. (2012). Negligible particle-specific antibacterial activity of silver nanoparticles. *Nano Lett*. 12 4271–4275 10.1021/nl301934w 22765771

