# Supplementary material for: Nanosilver induces a non-culturable but metabolically active state in Pseudomonas aeruginosa
Source: Front Microbiol. 2015 May 5;6:395. doi: 10.3389/fmicb.2015.00395 (PMC4419727; doi:10.3389/fmicb.2015.00395)
Supplement: Supplementary file 1 [file Table_1.DOCX]

***Supplementary Material***

**Nanosilver induces a non-culturable but metabolically active state in *Pseudomonas aeruginosa***

**Alexa Margareta Königs^1^*, Hans-Curt Flemming^1^, Jost Wingender^1^***

^1^Biofilm Centre, Department of Aquatic Microbiology, University Duisburg-Essen, Essen, Germany.

*** Correspondence:** Jost Wingender, Biofilm Centre, Department of Aquatic Microbiology, University Duisburg-Essen, Universitätsstrasse 5, 45141, Germany.

[jost.wingender@uni-due.de](mailto:jost.wingender@uni-due.de)

**Table 1.** Viability parameters of planktonic cells of *P. aeruginosa*.

| **Planktonic cells** | **colony  counts** | **total cell counts** | **cells with intact**  **cell membrane** | **FISH-positive  cells** | **ATP  (amol/cell)** |
| --- | --- | --- | --- | --- | --- |
| Control  without silver | 1.67 x 10^7^ | 7.60 x 10^8^ | 1.4 x 10^7^ | 4.04 x 10^8^ | 0.019 |
| Ag (AgNO_3_)  100 µg/ml | 0 | 9.06 x 10^8^ | 2.66 x 10^7^ | 3.42 x 10^8^ | 0.010 |
| Ag (AgNPs)  500 µg/ml | 0 | 8.55 x 10^8^ | 2.46 x 10^7^ | 4.63 x 10^8^ | 0.005 |
