# Supplementary material for: Nanosilver induces a non-culturable but metabolically active state in Pseudomonas aeruginosa
Source: Front Microbiol. 2015 May 5;6:395. doi: 10.3389/fmicb.2015.00395 (PMC4419727; doi:10.3389/fmicb.2015.00395)
Supplement: Supplementary file 2 [file Table_2.DOCX]

***Supplementary Material***

**Nanosilver induces a non-culturable but metabolically active state in *Pseudomonas aeruginosa***

**Alexa Margareta Königs^1^*, Hans-Curt Flemming^1^, Jost Wingender^1^***

^1^Biofilm Centre, Department of Aquatic Microbiology, University Duisburg-Essen, Essen, Germany.

*** Correspondence:** Jost Wingender, Biofilm Centre, Department of Aquatic Microbiology, University Duisburg-Essen, Universitätsstrasse 5, 45141, Germany.

[jost.wingender@uni-due.de](mailto:jost.wingender@uni-due.de)

**Table 2.** Viability parameters of silver exposed established biofilms of *P. aeruginosa*.

| **Biofilms** | **colony  counts** | **total cell counts** | **cells with intact**  **cell membrane** | **FISH-positive  cells** | **ATP  (amol/cell)** |
| --- | --- | --- | --- | --- | --- |
| Control  without silver | 7.66 x 10^6^ | 1.24 x 10^8^ | 1.0 x 10^7^ | 2.34 x 10^7^ | 0.330 |
| Ag (AgNO_3_)  100 µg/ml | 7.34 x 10^4^ | 9.38 x 10^7^ | 9.38 x 10^6^ | 6.30 x 10^7^ | 0.401 |
| Ag (AgNPs)  500 µg/ml | 1.21 x 10^5^ | 7.93 x 10^8^ | 1.69 x 10^7^ | 5.10 x 10^8^ | 0.057 |
