# Supplementary material for: Nanosilver induces a non-culturable but metabolically active state in Pseudomonas aeruginosa
Source: Front Microbiol. 2015 May 5;6:395. doi: 10.3389/fmicb.2015.00395 (PMC4419727; doi:10.3389/fmicb.2015.00395)

***Supplementary Material***

**Nanosilver induces a non-culturable metabolically active state in *Pseudomonas aeruginosa***

**Alexa Margareta Königs^1^*, Hans-Curt Flemming^1^, Jost Wingender^1^***

^1^Biofilm Centre, Department of Aquatic Microbiology, University Duisburg-Essen, Essen, Germany.

*** Correspondence:** Jost Wingender, Biofilm Centre, Department of Aquatic Microbiology, University Duisburg-Essen, Universitätsstrasse 5, 45141, Germany.

[jost.wingender@uni-due.de](mailto:jost.wingender@uni-due.de)

Figure 2. Absorbance spectra (300-600 nm) of AgNPs (20 µg/ml) in deionized water.


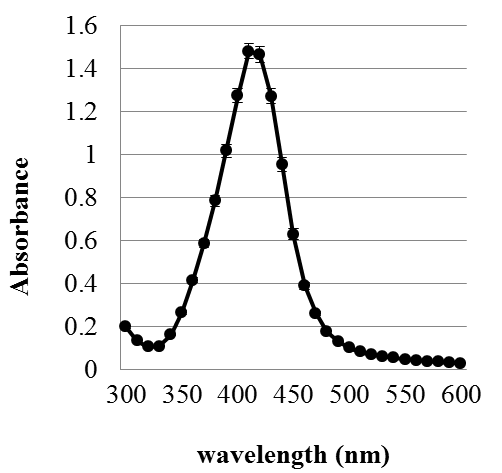

Supplement: Supplementary file 4 [file Table_4.DOCX]
